# Rotigotine and parkinsonism-hyperpyrexia syndrome

**DOI:** 10.1016/j.prdoa.2026.100447

**Published:** 2026-05-02

**Authors:** Shohei Watanabe, Ayano Hasuike, Hosho Morishita, Koji Yagi, Eiko Fujiwara, Daisuke Yamanaka, Shuhei Kasama, Masataka Igeta, Takashi Kimura

**Affiliations:** a Department of Neurology, Hyogo Medical University, 1-1 Mukogawa-cho, Nishinomiya City, Hyogo, Japan; b Department of Biostatistics, Hyogo Medical University, 1-1 Mukogawa-cho, Nishinomiya City, Hyogo, Japan

**Keywords:** Parkinson’sdisease, Parkinsonism-Hyperpyrexiasyndrome, Rotigotine

## Abstract

**Introduction:**

Parkinsonism–hyperpyrexia syndrome (PHS) is a potentially fatal complication of Parkinson’s disease (PD) often triggered by antiparkinsonian medication discontinuation or reduction. Rotigotine, a transdermal dopamine agonist, provides continuous drug delivery and can be administered even when oral administration is difficult. However, the association between rotigotine use and development of PHS remains unclear.

**Methods:**

We retrospectively reviewed 166 episodes of emergency hospitalization for PD at Hyogo Medical University Hospital (2010–2024). After excluding 19 episodes with insufficient data, 147 were analyzed. Thirty episodes met the diagnostic criteria for PHS (PHS group), and 117 episodes did not (non-PHS group). Clinical characteristics, antiparkinsonian medications, and precipitating events during hospitalization were assessed. A multivariable logistic regression analysis was performed using age, sex, disease duration, and rotigotine administration as covariates.

**Results:**

The PHS group had a shorter disease duration, lower modified Hoehn and Yahr stages, and a lower levodopa equivalent dose than the non-PHS group. Poor medication compliance was the most common precipitating factor among PHS cases (n = 11, 36.7%). Rotigotine was administered in five PHS and 41 non-PHS episodes. In the multivariable analysis, age was significantly negatively associated with the development of PHS (odds ratio [OR], 0.894; 95% confidence interval [CI], 0.840–0.952; p = 0.0005). And rotigotine administration showed a tendency to suppress the development of PHS (OR, 0.353; 95% CI, 0.121–1.031; p = 0.0569).

**Conclusion:**

Although not statistically significant, rotigotine use tends to be associated with a lower incidence of PHS. Maintaining good medication adherence is critical for preventing PHS, and we consider that rotigotine may be a useful therapeutic option.

## Highlights

• Rotigotine showed a tendency to suppress the development of PHS.
• Maintaining good medication compliance is crucial for suppressing PHS in PD.
• Rotigotine is a once-daily transdermal patch that promotes medication compliance.

## 1 Introduction

Parkinsonism-hyperpyrexia syndrome (PHS) is a potentially life-threatening disorder in patients with Parkinson’s disease (PD). PHS is characterized by hyperthermia, prominent rigidity, disturbance of consciousness, autonomic dysfunction, and elevated serum creatine kinase (CK) [1]. The main triggering event of PHS is antiparkinsonian medication discontinuation or reduction. Other triggering events are also recognized, such as infections, motor fluctuations, and anorexia, which can also unintentionally interrupt oral drug intake [2], [3], [4]. Additionally, it has been reported that PHS is prone to develop in elderly patients with PD with a long disease duration and is predominantly found in males [5], [6].

New drugs for the treatment of PD were recently developed one after another. In particular, rotigotine transdermal patches (dopamine agonists D_1_-D_5_) continuously deliver the drug for over 24 h, with the additional advantage of being administered even when oral drug intake is difficult [7], [8]. However, the relationship between the onset of PHS and antiparkinsonian medications has not been sufficiently investigated to date.

In this study, we compared the clinical characteristics, including details of antiparkinsonian drugs, between PHS and non-PHS cases during the emergency hospitalization of patients with PD in our institution. In particular, we investigated whether rotigotine suppressed the onset of PHS.

## 2 Methods

### 2.1 Selection of patients and clinical evaluation

We retrospectively examined the medical records of 166 episodes of emergency hospitalization of 117 patients with PD at Hyogo Medical University Hospital from January 2010 to December 2024. We confirmed the diagnosis of PD in these patients using the Movement Disorder Society clinical diagnostic criteria [9]. We excluded 19 episodes with insufficient medical information and investigated the medical records of the remaining 147 episodes (114 patients).

Since widely accepted diagnostic criteria for PHS have not yet been established, we adopted the modified Levenson diagnostic criteria for neuroleptic malignant syndrome to diagnose PHS (Table 1) [2], [10]. As per Levenson’s criteria, 30 episodes (28 patients) met the PHS diagnostic criteria (PHS group), and 117 episodes (86 patients) that did not meet the criteria (non-PHS group). Two patients experienced recurrent episodes of PHS. We then assessed the following clinical characteristics based on the medical records: sex, age, PD duration, modified Hoehn & Yahr (m H&Y) stage (both on and off states), levodopa (LD) dose, LD equivalent dose (LED) [11], and details of antiparkinsonian medications. Additionally, we assessed the precipitation events of emergency hospitalization in both the PHS and non-PHS groups. “Medication withdrawal/reduction” was defined as being due to the healthcare provider's instructions. On the other hand, “poor medication compliance” was defined as a situation in which a patient fails to adhere to the prescribed medication regimen due to their own actions. Medication compliance was confirmed through interviews with the patients and/or their family.

**Table 1:** PHS diagnostic criteria.

Major manifestations Fever Exacerbation of parkinsonism Elevated CK Minor manifestations Tachycardia Abnormal blood pressure Tachypnea Altered consciousness Diaphoresis Leukocytosis Enrolment criteria The presence of all three major manifestations or two major + four minor ones

Abbreviations: CK, creatine kinase; PHS, Parkinsonism-Hyperpyrexia syndrome.

This study was approved by the Ethics Committee of the Hyogo Medical University (approval number: 4211).

### 2.2 Statistical analysis

Continuous variables were summarized as mean ± standard deviation (SD). Categorical variables were presented as frequencies and percentages within each group. Between-group differences were descriptively assessed. Based on a previous literature review [5], we selected the following factors for multivariable analysis to evaluate the effect of rotigotine on PHS: age, PD duration, sex, and rotigotine administration. Multivariable logistic regression based on generalized estimating equations was performed to estimate odds ratios (ORs) for the development of PHS. Because some patients contributed more than one hospitalization episode, within-patient correlation was accounted for by specifying patient-level clustering with an exchangeable working correlation structure. Wald-type confidence intervals (CIs) based on robust sandwich standard errors were calculated for regression coefficients, which were exponentiated to obtain ORs. The threshold for statistical significance was set at p < 0.05. The analyses were performed using EZR (Saitama Medical Center, Jichi Medical University, Japan), a graphical user interface for R (The R Foundation for Statistical Computing, Vienna, Austria) [12], and SAS software (SAS Institute, Cary, NC, USA).

## 3 Results

### 3.1 Clinical characteristics

The clinical characteristics of the PHS and non-PHS groups are summarized in Table 2. The age, PD duration, and m H&Y stage of the PHS group were lower than those of the non-PHS group. Furthermore, the PHS group had lower LD and LED. Rotigotine was administered in 5 PHS (dose:10.8 ± 2.5 mg/day, duration of administration:10 ± 6.7 months) and 41 non-PHS (dose: 13.1 ± 5.4 mg/day, duration of administration: 26.7 ± 18.9 months) cases. Eight patients prescribed rotigotine experienced two recurrent episodes and one patient prescribed rotigotine experienced three recurrent episodes of emergency hospitalization due to causes other than PHS (non-PHS cases). One patient prescribed rotigotine experienced a recurrence of PHS. The details of 5 PHS cases prescribed rotigotine are summarized in Table 3.

**Table 2:** Patient characteristics.

	PHS group	Non-PHS group
N	30	117
Sex (male)	11 (36.7)	55 (47.0)
Age, years	69.8 ± 7.7	74.8 ± 7.5
PD duration, years	8.5 ± 6.8	11.5 ± 7.0
m H & Y stage (ON state)	3.9 ± 0.6	4.3 ± 0.7
m H & Y stage (OFF state)	4.0 ± 0.6	4.5 ± 0.6
LD dose, mg/day	411.7 ± 165.9	507.8 ± 294.7
LED, mg/day	722.0 ± 348.0	837.3 ± 516.1
Coadministrated anti-PD drugs		
Bromocriptine	none	1 (0.9)
Pergolide	2 (6.7)	1 (0.9)
Cabergoline	1 (3.3)	1 (0.9)
Pramipexole	9 (30.0)	27 (23.1)
Ropinirole oral	2 (6.7)	14(12.0)
Ropinirole patch	1 (3.3)	4 (3.4)
Rotigotine	5 (16.7)	41 (35.0)
Selegiline	7 (23.3)	29 (24.8)
Rasagiline	none	9 (7.7)
Safinamide	2 (6.7)	1 (0.9)
Entacapone	11 (36.7)	29 (24.8)
Opicapone	2 (6.7)	5 (4.3)
Amantadine	5 (16.7)	12 (10.3)
Trihexyphenidyl	2 (6.7)	5 (4.3)
Biperidene	2 (6.7)	1 (0.9)
Droxidopa	2 (6.7)	9 (7.7)
Zonisamide	8 (26.7)	34 (29.1)
Istradefylline	2 (6.7)	18 (15.4)

Abbreviations: LD, levodopa; LED, levodopa equivalent dose; m H & Y, modified Hoehn & Yahr; PD, Parkinson’s disease; PHS, Parkinsonism-Hyperpyrexia syndrome.

Continuous variables are presented as mean ± standard deviation (SD).

Categorical variables are presented as n (%).

**Table 3:** Characteristics of PHS cases prescribed rotigotine.

Patient number	Age	Sex	PD duratuion(years)	Body temperture (axillary, ℃)	CK(U/L)	Leukocytes (/µL)	rotigotine dose(mg/day)	rotigotine duration (months)	other anti-PD drugs (mg/day)	precipitating events for PHS
1	65	F	2	37.8	6450	6710	9	5	LD 500	fall and difficulty moving
	66		3	37.8	2494	5120	13.5	10	LD 600	fall and difficulty moving
2	75	F	10	40	4642	14,440	9	12	LCE 300, AMA 300, ZNS 25	poor medication compliance
3	72	F	28	38.9	610	10,980	9	3	LCE 200, AMA 300, SEL 5, PRA 1	poor food intake and difficulty moving
4	59	M	23	38.4	29,793	11,120	13.5	20	LD 450, AMA 200, SEL 5, BIP 1.5, CAB 2.5	poor medication compliance

Abbreviations: AMA, amantadine; BIP, biperiden; CAB; cabergoline; CK, creatine kinase; LCE, levodopa carbidopa hydrate entacapone; LD, levodopa; PD, Parkinson’s disease; PHS, Parkinsonism-Hyperpyrexia syndrome; PRA, pramipexole; SEL, selegiline; ZNS, zonisamide.

The ropinirole patch, another dopamine agonist transdermal patch, was administered to only one PHS and four non-PHS cases. Neither bromocriptine nor rasagiline was prescribed to the PHS group.

### 3.2 Events leading to emergency hospitalization

The events leading to emergency hospitalization are summarized in Table 4. In the PHS group, poor medication compliance was particularly noticeable (n = 11, 36.7%). Meanwhile, the most common event in the non-PHS group was infection (n = 59, 50.4%), especially that of the respiratory tract (n = 38, 32.5%).

**Table 4:** Events leading to emergency hospitalization.

	PHS group	Non-PHS group
N	30	117
Medication withdrawal/reduction	1 (3.3)	2 (1.7)
Because of hallucination	1 (3.3)	none
skin disorders	none	1 (0.9)
nausea	none	1 (0.9)
Poor medication compliance	11 (36.7)	1 (0.9)
Infection	5 (16.7)	59 (50.4)
Respiratory tract	1 (3.3)	38 (32.5)
Urinary tract	4 (13.3)	11 (9.4)
Other region	none	10 (8.5)
Motor fluctuations alone	1 (3.3)	23 19.7)
Fall/fracture	6 (20.0)	7 (6.0)
Anorexia/poor food intake	2 (6.7)	2 (1.7)
Dysphagia	2 (6.7)	none
Choke	none	4 (3.4)
Dehydration	1 (3.3)	4 (3.4)
Ileus	none	2 (1.7)
Altered consciousness	none	3 (2.6)
Hallucination	none	2 (1.7)
Others	1 (3.3)	8 (6.8)

Abbreviations: PHS, Parkinsonism-Hyperpyrexia syndrome.

Values are presented as n (%).

### 3.3 Association between PHS and rotigotine

In the multivariable analysis, age was significantly negatively associated with the development of PHS (OR, 0.894, 95% CI, 0.840–0.952, p = 0.0005). And rotigotine administration showed a tendency to suppress the development of PHS, although this result did not reach significance (OR, 0.353; 95% CI, 0.121–1.031; p = 0.0569) (Table 5).

**Table 5:** Results of multivariable analyses of clinical factors for the onset of PHS.

Variable	Multivariable OR (95% CI)	*p*
Sex (Male/Female)	0.479 (0.186–1.237)	0.1286
Age, years	0.894 (0.840–0.952)	0.0005
PD duration, years	0.929 (0.846–1.020)	0.1201
Administration of Rotigotine (Yes/No)	0.353 (0.121–1.031)	0.0569

Abbreviations: CI, confidence interval; OR, odds ratio; PD, Parkinson’s disease; PHS, Parkinsonism-hyperpyrexia syndrome.

## 4 Discussion

Although not statistically significant, rotigotine tended to be negatively associated with the development of PHS in this study. We hypothesize that rotigotine may have a protective effect against PHS. Rotigotine is a non-ergolinic dopamine agonist (D_1_-D_5_) administered via a transdermal patch once a day. Because of the transdermal formulation, rotigotine is absorbed through the skin over 24 h, making it easier to maintain stable plasma concentrations compared to other dopamine agonists administered orally several times a day [7], [13]. Additionally, even when oral administration is not possible or intestinal motility disorders cause poor drug absorption, rotigotine shows a certain degree of efficacy. In our study, the most common precipitation event in PHS was poor medication compliance. Treatment of advanced PD often involves multiple medications, and it is critical to maintain good medication compliance for patients with PD. As rotigotine is a transdermal patch drug administered once daily, it can encourage better medication compliance than can other oral antiparkinsonian drugs. Therefore, we speculated that the use of rotigotine could prevent the onset of PHS.

Although the discontinuation or dose reduction of antiparkinsonian drugs was reported to be the most common cause of PHS in previous studies [2], [6], only one such case was found in this study (medication withdrawal/reduction). As the risk of PHS has become widely recognized, abrupt reductions or discontinuations of antiparkinsonian medication by healthcare providers may have decreased. Nevertheless, the risk of sudden dose reduction or discontinuation by patients with PD themselves (particularly those with comorbid dementia or poor medication compliance) remains, and the people around them are often unaware of the discontinuation of medication. Transdermal patch drugs, such as rotigotine, are labeled with the date of administration and are useful for verifying medication compliance. These characteristics of rotigotine may play a role in preventing PHS onset. However, we found 5 PHS cases prescribed rotigotine. If a PD patient takes a large dose of LD and/or takes multiple antiparkinsonian drugs, we may need to be cautious of the development of PHS.

This study has some limitations. First, only patients from one institution (a university hospital) were involved, and the number of patients was not large. Because the sample size was small, we may not have been able to find significant correlation between rotigotine administration and the development of PHS. In contrast to previous reports [5], [6], our study showed that age was negatively associated with PHS. It is also possible that younger patients manage their medication without support from those around them, which may ultimately lead to poor medication compliance. The small sample size and/or facility bias (one university hospital) may have caused these conflicting results. Owing to the small sample size, it was also necessary to narrow down the factors for multivariable analysis. Generally, the m H&Y stage, LD dose, and LED are associated with PD duration. Therefore, we did not include these three factors in the multivariable analysis. Furthermore, the ropinirole transdermal patch, another transdermal dopamine agonist, was not fully investigated because only a few patients took it in this study. A comparison of rotigotine and ropinirole transdermal patch will be necessary in the future. Second, we may not have accurately analyzed medication compliance in these patients immediately before emergency hospitalization because this was a retrospective study. Further prospective multicenter studies with larger numbers of patients with both PD and PHS are necessary.

In conclusion, maintaining good medication compliance is crucial for suppressing the onset of PHS in patients with PD, and we consider that rotigotine may be a good option. Physicians should consider details of antiparkinsonian medications from the perspective of improving medication compliance to suppress the onset of PHS.

## CRediT authorship contribution statement

**Shohei Watanabe:** Writing – original draft, Validation, Project administration, Methodology, Investigation, Data curation, Conceptualization. **Ayano Hasuike:** Data curation. **Hosho Morishita:** Data curation. **Koji Yagi:** Data curation. **Eiko Fujiwara:** Data curation. **Daisuke Yamanaka:** Data curation. **Shuhei Kasama:** Data curation. **Masataka Igeta:** Validation, Methodology, Formal analysis. **Takashi Kimura:** Validation, Supervision, Project administration.

## Funding

This research did not receive any specific grant from funding agencies in the public, commercial, or not-for-profit sectors.

## Declaration of competing interest

The authors declare that they have no known competing financial interests or personal relationships that could have appeared to influence the work reported in this paper.
